# Molecular Dynamics Simulation Reveals the Selective Binding of Human Leukocyte Antigen Alleles Associated with Behçet's Disease

**DOI:** 10.1371/journal.pone.0135575

**Published:** 2015-09-02

**Authors:** Sirilak Kongkaew, Pathumwadee Yotmanee, Thanyada Rungrotmongkol, Nopporn Kaiyawet, Arthitaya Meeprasert, Toshikatsu Kaburaki, Hiroshi Noguchi, Fujio Takeuchi, Nawee Kungwan, Supot Hannongbua

**Affiliations:** 1 Program in Biotechnology, Faculty of Science, Chulalongkorn University, Bangkok, Thailand; 2 Department of Chemistry, Faculty of Science, Ramkhamhaeng University, Bangkok, Thailand; 3 Department of Biochemistry, Faculty of Science, Chulalongkorn University, Bangkok, Thailand; 4 Ph.D. Program in Bioinformatics and Computational Biology, Faculty of Science, Chulalongkorn University, Bangkok, Thailand; 5 Computational Chemistry Unit Cell, Department of Chemistry, Faculty of Science, Chulalongkorn University, Bangkok, Thailand; 6 Department of Ophthalmology, University of Tokyo, Tokyo, Japan; 7 School of Pharmaceutical Sciences, University of Shizuoka, Shizuoka, Japan; 8 Department of Internal Medicine, Faculty of Medicine, University of Tokyo, Tokyo, Japan; 9 Department of Chemistry, Faculty of Science, Chiang Mai University, Chiang Mai, Thailand; Wake Forest University, UNITED STATES

## Abstract

Behçet’s disease (BD), a multi-organ inflammatory disorder, is associated with the presence of the human leukocyte antigen (HLA) HLA-B*51 allele in many ethnic groups. The possible antigen involvement of the major histocompatibility complex class I chain related gene A transmembrane (MICA-TM) nonapeptide (AAAAAIFVI) has been reported in BD symptomatic patients. This peptide has also been detected in HLA-A*26:01 positive patients. To investigate the link of BD with these two specific HLA alleles, molecular dynamics (MD) simulations were applied on the MICA-TM nonapeptide binding to the two BD-associated HLA alleles in comparison with the two non-BD-associated HLA alleles (B*35:01 and A*11:01). The MD simulations were applied on the four HLA/MICA-TM peptide complexes in aqueous solution. As a result, stabilization for the incoming MICA-TM was found to be predominantly contributed from van der Waals interactions. The P2/P3 residue close to the N-terminal and the P9 residue at the C-terminal of the MICA-TM nonapeptide served as the anchor for the peptide accommodated at the binding groove of the BD associated HLAs. The MM/PBSA free energy calculation predicted a stronger binding of the HLA/peptide complexes for the BD-associated HLA alleles than for the non-BD-associated ones, with a ranked binding strength of B*51:01 > B*35:01 and A*26:01 > A*11:01. Thus, the HLAs associated with BD pathogenesis expose the binding efficiency with the MICA-TM nonapeptide tighter than the non-associated HLA alleles. In addition, the residues 70, 73, 99, 146, 147 and 159 of the two BD-associated HLAs provided the conserved interaction for the MICA-TM peptide binding.

## Introduction

Behçet’s disease (BD) has caused recurrent inflammation of symptom complex in genital and oral ulceration, inflammatory lesion of central nervous systems, gastrointestinal tract and eyes leading to blindness [1]. This disease is usually reported in adults aged between 18 to 40 year-olds and presents a higher mortality risk in males than females [2]. A high epidemiology of BD is commonly found in Japan, Turkey, China and the Middle East and the Mediterranean countries [3]. The systemic inflammatory disease is characterized by recurrent exacerbations and spontaneous remissions with varying healing times among patients, and the disease course usually becomes several years or more. On the ocular disease, recurrent and often bilateral episodes of acute exacerbations of intraocular inflammation (ocular attacks) in the anterior chamber (iridocyclitis) normally occur with or without posterior involvement (retinal vasculitis, hemorrhages, exudates, retinal vein occlusion and optic neuritis). Recurrent ocular attacks of posterior involvement lead to poor visual prognosis [4]. Intestinal and neurological diseases are serious complications of BD and can severely deteriorate the patients’ psychosomatic status. Because BD can induce severe damage in the eyes and other organs, it is important to clarify the pathogenesis of this disease and to develop a novel treatment based on the pathogenesis.

The exact etiology of this disease has not been clarified yet, but it is probably mediated by a combination of excessive immune reactions, genetics factors [5], immune reactions against infectious agents [6], heat shock proteins [7], oxidative stress [8] and environmental factors. Indeed, several inflammatory cytokines involved in acute inflammatory reactions, such as interleukin (IL)-6, tumor necrosis factor-*α* and IL-8 might play a role in the pathogenesis of this disease [9]. In addition, genetic factors, including the human leukocyte antigen (HLA)-B51 alleles and especially the HLA-B*51:01 allele is strongly associated with BD across different susceptible ethnic groups [10, 11]. Recently, a meta-analysis of 78 studies involving 4,800 cases of BD and 16,289 controls reported the pooled odds ratio (OR) of HLA B51/B5 carriers to develop BD compared to non-carriers (OR = 5.78 95%, CI = 5.00–6.67) [12].

The notion of an environmental trigger of BD in patients with genetic susceptibilities has long been advocated. Several infectious agents have been investigated, especially bacteria (*Streptococcus*, *Mycoplasma* and *Helicobacter pylori*) and viruses (Herpes simplex virus 1 and 2, Hepatitis virus and Parvovirus B19) [13]. The autoimmunity against bacterial and human heat-shock proteins (HSP) was also speculated as a trigger for BD, because autoantibodies against HSP65 and HSP60 have been reported in BD patients [14].

On the other hand, the genetic association of the major histocompatibility complex (MHC) class I chain related to gene A (MICA) with BD has been reported in various ethnic groups [15, 16], and a strong association of the polymorphism in the transmembrane region of MICA (MICA*009) with BD was observed [17, 18]. The self nonapeptide of AAAAAIFVI located on the MICA transmembrane region (MICA-TM), induced autoreactive CD8+ cytotoxic T lymphocytes in BD patients with HLA-B*51 [19]. Accordingly, the MICA-TM peptide is one of the candidate bechetogenic epitopes [17, 19], where the self-antigen AAAAAIFVI could function as the antigen that triggers the T-cell receptor and develops to an autoimmune reaction [19–21]. The MICA is usually expressed in fibroblasts, monocytes, epithelial cells and endothelial cells in a stress-dependent manner, and plays a key role in initiating the antibody-dependent rejection of organ-transplants [22–24].

**Fig 1:**
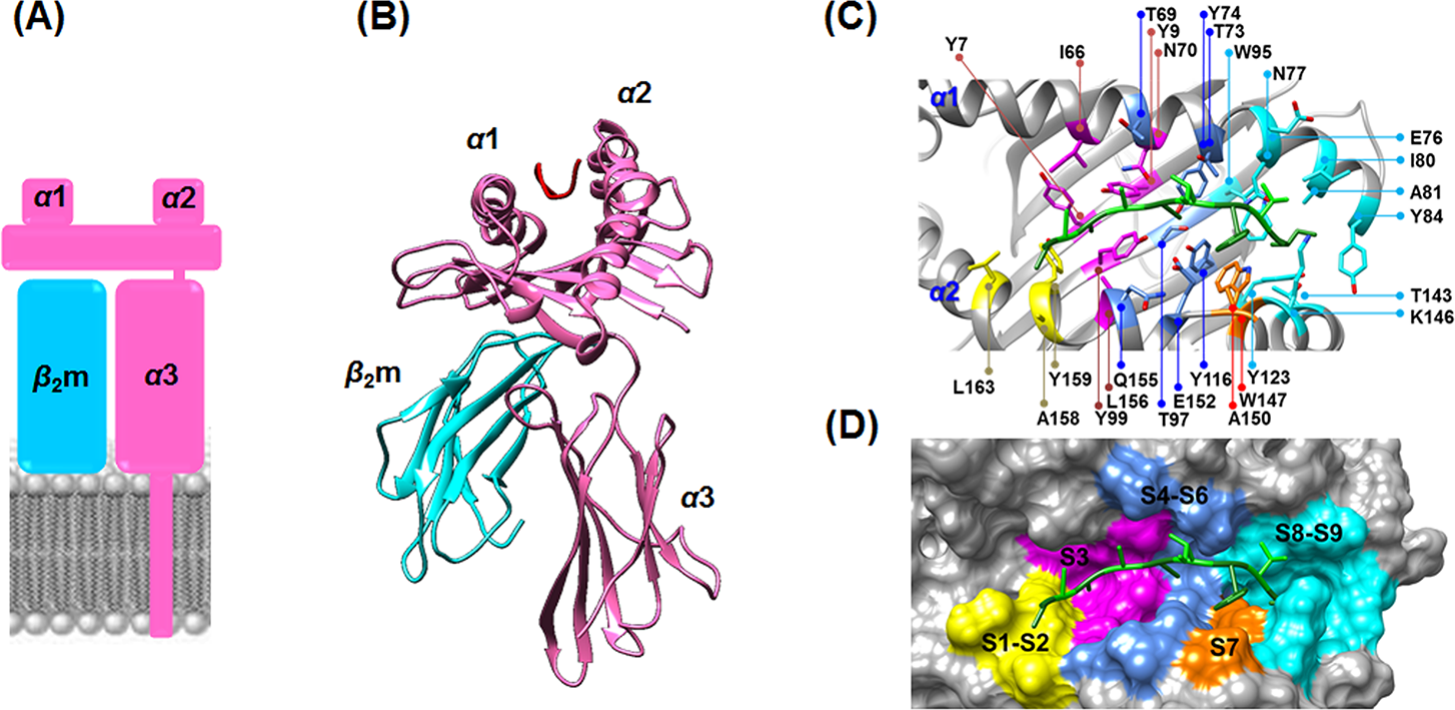
Structural basis of HLA class I. (A) Schematic model of HLA buried in the transmembrane. (B) HLA (pink) contains the *α*1 and *α*2 subdomains that contribute to the peptide binding groove, while *α*3 is the C-terminal domain in complex with *ß*_2_-microgluobulin (*ß*_2_m) as a noncovalently supported protein (cyan). (C) Ribbon and (D) van der Waals surface representations of the MICA-TM nonapeptide (green stick model) occupied in the peptide binding sub-sites (S1–S9, shaded by different colors) of HLA-B*51:01.

The HLA molecules are human equivalents of the MHC found in most vertebrates [25]. They are controlled by genes located at the terminal region at band p21.3 on the chromosome 6. Normally, the HLA molecules play a key role as a guard to protect cells in immune recognition. The HLAs are categorized into three different classes (I, II and II), with HLA class I further divided into three main genes(HLA-A,-B and-C) [26]. The HLA alleles associated with BD involve the HLA class I genes HLA-A and HLA-B that importantly function to bind and send short foreign antigens from within the cell to the T-cell receptor (TCR) of CD8+ glycoprotein cells (cytotoxic or killer T-cells) [27]. A digested antigen peptide consisting of 8–13 amino acid residues in length (most commonly 9 to 10 residues) is specifically bound within the peptide-binding groove located on the HLA surface [28, 29]. The binding groove is constructed from the eight *ß*-strands and *α*1 and *α*2 helixes, while *α*3 and *ß*_2_-microglobulin (*ß*_2_m) are the floor and non-covalent supported units, respectively, as shown in Fig 1 [28]. The different diversity of residues present in the binding-cleft markedly affects the specific binding to the antigen peptide and pathogenesis. The residues at or close to the two ends of the peptide, P2/P3 and P9 residues for the MICA-TM nonapeptide, generally interact with the binding groove of HLA class I and serve as an anchor, whilst the central peptide oozes up out of the groove for direct interaction with the TCR [30, 31]. However, peptides longer than nine residues and up to 13 residues can be recognized by the HLA class I genes through “the middle-out loop shape form” [30], where the residues at or close to the peptide terminals are well occupied in their sub-sites [29].

The association of BD with the HLA-B*51:01 allele in various ethnic groups in Japan and Korea has been frequently observed [3], while the HLA-A*26:01 allele was detected in BD patients in Greece, Japan and Taiwan [32–34]. Some possible candidate genes for BD have been studied in various HLA-A and-B alleles [35]. Recently, in Japanese uveitis patients, BD was associated with the HLA-A*26:01 allele at a 37.5% phenotype frequency more than the controls, and so HLA-A*26:01 is a possible marker as a susceptible allele for ophthalmic BD in Japanese ethnics [36]. By meta-analysis and positive pathergy tests of the Japanese data, the BD clinical manifestations of uveitis, skin lesions and arthritis, and genital ulcers were found to be significantly associated with the HLA-A*26:01, A*02:07 and A*30:04 alleles, respectively [37]. Currently there is no treatment (e.g. vaccine) against BD based upon the HLA recognition antigens despite the apparent restricted association with specific alleles, since the individual risk factors with HLA are unknown. Therefore, we aimed to investigate the link between BD and two specific HLA alleles associated with BD (HLA-A*26:01 and HLA-B*51:01) in terms of their binding affinity to the MICA-TM peptide using MD simulations, in comparison with two HLA alleles not associated with BD (HLA-A*11:01 and HLA-B*35:01). The molecular understanding of the specific binding of the MICA-TM peptide with the HLA alleles associated with the disease may be useful for therapeutic vaccine design.

## Materials and Methods

### Structure preparation

The starting structures of three of the HLA class I alleles were taken from the Protein Data Bank, being entries 1E27 [38] for B*51:01 with the HIV-1 epitope at 2.20 Å, 1X7Q [39] for A*11:01 with the SARS nucleocapsid at 1.45 Å, and 1A9E [40] for B*35:01 with the EVB peptide at 2.5 Å (S1 Table). That for A*26:01 was built from homology modeling using the above A*11:01/SARS nucleocapsid structure as a template and the amino acid sequence of A*26:01 from GenBank (accession no. AAA03720) [41], which has 94.3 and 96.6% amino acid identity and similarity to A*11:01, respectively. The HLA/MICA-TM complexes were constructed by changing the original peptide within the X-ray structure to be consistent with the nine amino acids of the antigenic MICA-TM peptide (AAAAAIFVI) using the align sequence profiles module implemented in the Discovery studio 2.5 (Accelrys, Inc.).

### MD simulation

The four studied HLA/MICA-TM complexes were investigated by molecular dynamics (MD) simulations using the AMBER 10 software package [42] with the ff03 force field. The missing atoms were added using the LEaP module [43]. The protonation state of all possible charged residues (arginine, lysine, histidine, aspartate and glutamate) in HLA-allele complexes was assigned at pH 7.0 by PROPKA server [44]. The total charges with negative value of the HLA/MICA-TM complexes were randomly neutralized by Na^+^ counterions (2, 1, 1 and 2 ions for the B*51:01, B*35:01, A*26:01 and A*11:01 systems, respectively). Afterwards, the individual complex was solvated by TIP3P [45] water molecules leading to approximately 65,000 atoms in total. The dimensions of the simulation box used for all the systems were 86 × 90 × 88 Å^3^. The periodic boundary condition with the NPT ensemble and a simulation time step of 2 fs was used. All energy minimizations and MD simulations were performed using the SANDER module of AMBER 10. All bonds and angles concerned to hydrogen atoms were constrained by algorithm of SHAKE [46]. The long-range electrostatic interactions were treated by particle mesh Ewald method and the non-bonded interactions with a cutoff distance of 12 Å were considered [47]. All MD simulations were run with a 12 Å residue-based cutoff for non-bonded interactions and the particle mesh Ewald method was applied for an adequate treatment of long-range electrostatic interactions [48]. Each system was subjected to the four stages of the restrained MD simulations at 298 K with force constants of 10, 7.5, 5 and 2.5 kcal∙mol^-1^∙Å^2^ for 500 ps in each stage accordingly. These subsequent steps could allow the peptide to adapt its geometry and orientation from the initial model to fit better within the peptide binding groove. Then, the constraints were completely removed and fully unrestrained MD simulations were performed until 50 ns. The convergences of energies, temperature, and global root mean-square displacement (RMSD) were used to verify the stability of the systems. The MD trajectories were collected every 0.2 ps from the production phase for further analysis.

## Results and Discussion

### Stability of the HLA/MICA-TM complexes

To study the structural stability of the four MD simulations, the RMSDs for all atoms of the four HLA alleles (B*51:01, B*35:01, A*26:01 and A*11:01 alleles) complexed with the MICA-TM peptide compared with those of the starting structures were monitored along 50 ns of simulation time using the PTRAJ module implemented in AMBER10 package [49]. The RMSD fluctuations of each complex and its structural components (binding groove and *ß*_2_-microglobulin) and MICA-TM peptide, were plotted in S1 Fig. The RMSD values quickly increased until ~5 ns and then reached a plateau except for the HLA-B*51:01/MICA-TM complex where the RMSD value continuously increased over the first ~15 ns. The MICA-TM in HLA-B*51:01 system had a low fluctuation around 1 Å, and suddenly at 23 ns, it jumped up to 2 Å until the end simulation. The RMSD increasing caused from the non-polar of MICA-TM peptide change orientation to hydrophobic region within pocket. Although the whole HLA/MICA-TM complex for the two BD-associated HLAs (B*51:01 and A*26:01) fluctuated a great deal, their binding groove and the incoming MICA-TM peptide demonstrated a rather low level of fluctuation. All systems seemed to reach equilibrium after 25 ns with ~1 Å of RMSD fluctuation, and so the MD trajectories from 25 to 50 ns (the production phase) were used for analysis.

### Regional flexibility

**Fig 2:**
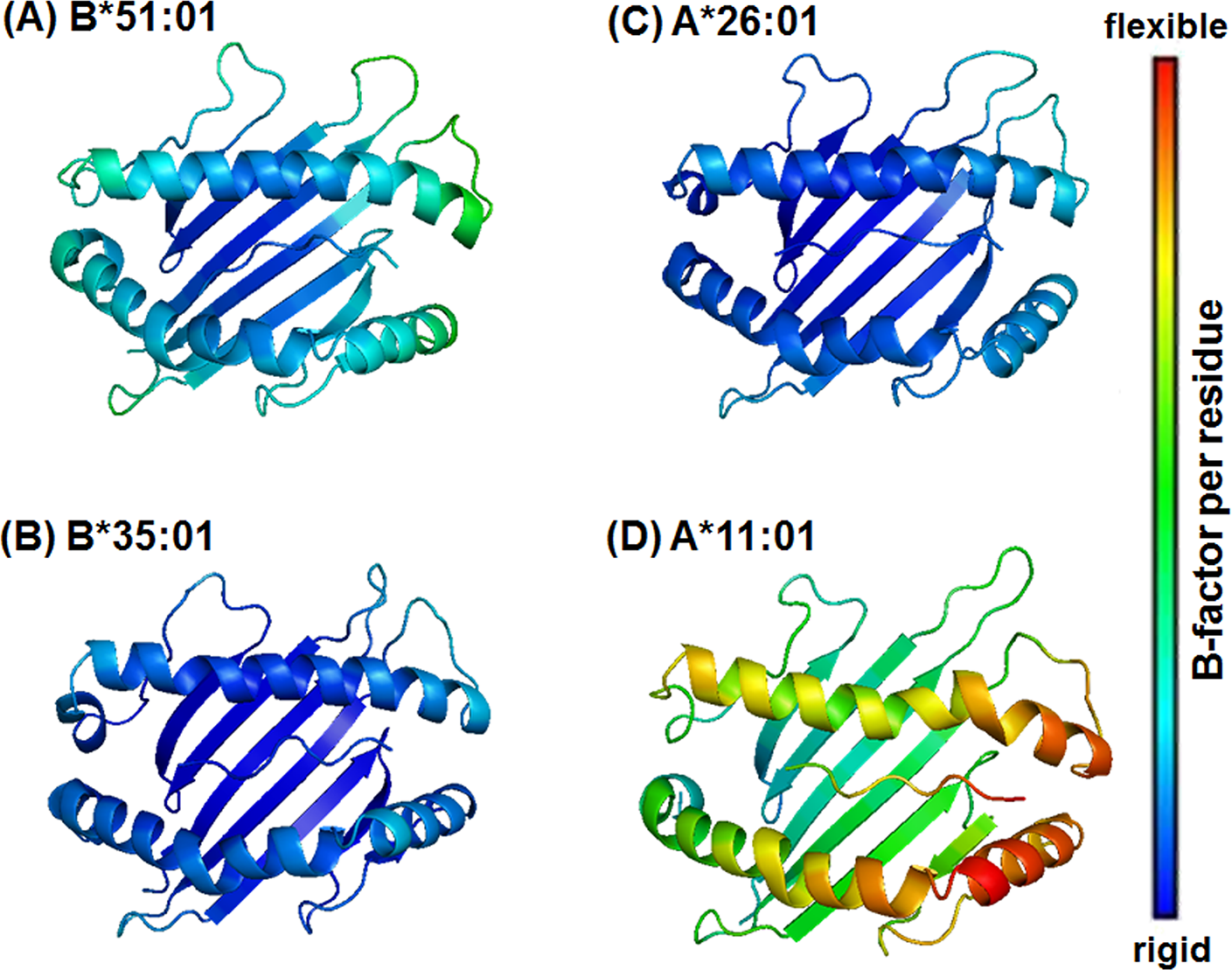
Structural flexibilities of the HLA alleles bound with the MICA-TM peptide. Structural flexibilities were evaluated by B-factor. The ribbon color changes from blue (rigid) to red (flexible) to represent a low to high protein flexibility. Note that for clarity only the binding groove structure and the MICA-TM peptide are shown.

The backbone flexibility of the HLA-B*51:01, B*35:01, A*26:01 and A*11:01 complexes with MICA-TM were investigated by B-factor calculation over the last 25 ns trajectories (Fig 2). Note that high flexibility of protein was displayed in red and *vice versa* in blue. Among the four systems, the HLA-A*11:01 allele showed the highest degree of protein flexibility in the region of peptide binding groove and in particular at the S7–S9 sub-sites (defined in Fig 1D), whereas the other HLA alleles were likely to have similar degree of flexibility. This might be due to the strong intramolecular interactions of either the *α*1 or *α*2 domains and the intermolecular hydrogen bonds (H-bonds) between the C-terminal of the MICA-TM peptide and the binding residues of the three HLAs (B*51:01, B*35:01 and A*26:01 alleles), which is discussed in a later section.

### Per-residue decomposition (DC) energy

**Fig 3:**
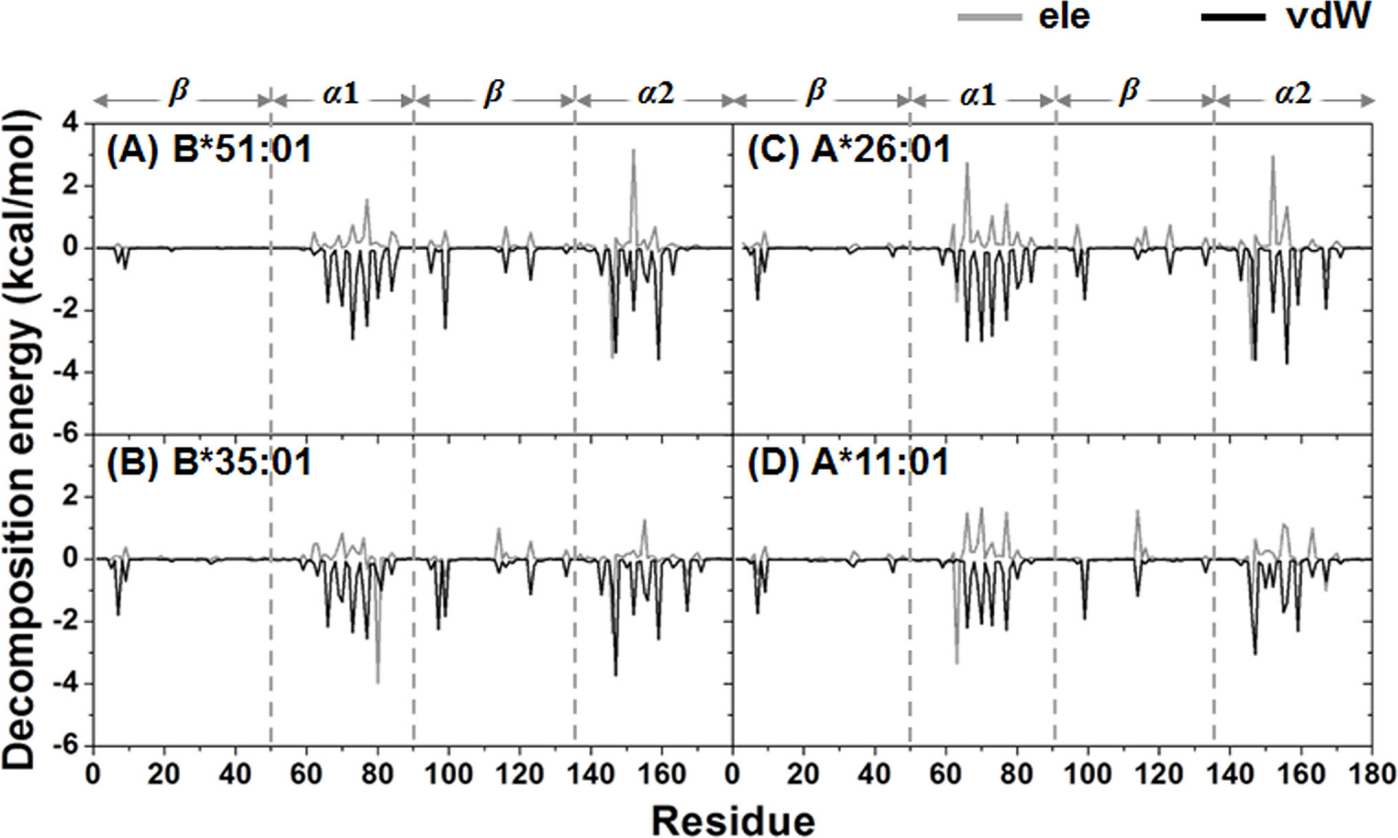
Decomposition energy per HLA residue fingerprint plots. The HLA contribution to the MICA-TM binding is shown in terms of the electrostatic (ele) and van der Waals (vdW) interactions.

**Fig 4:**
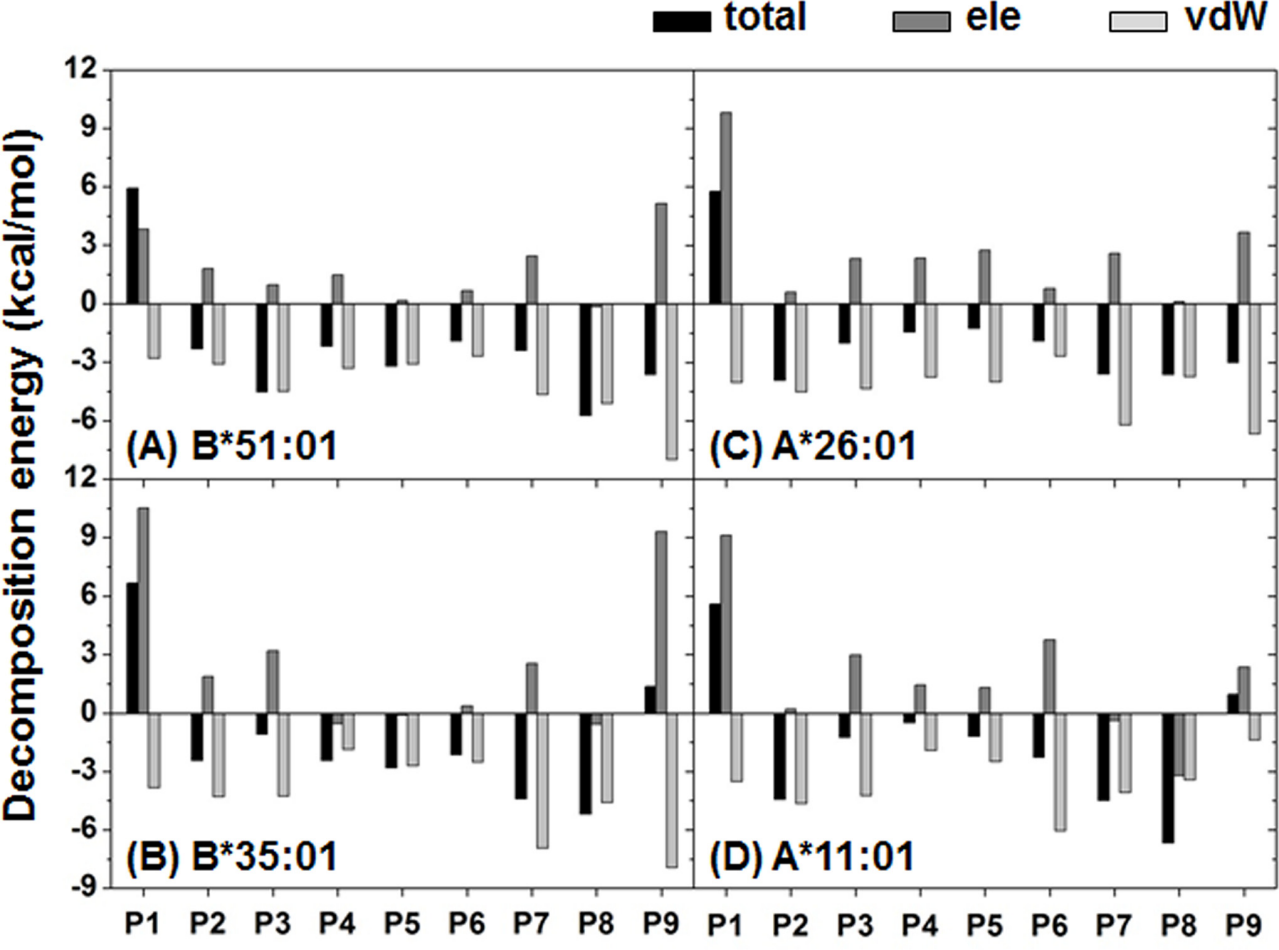
Averaged decomposition energy contributions in HLA binding to MICA-TM. Per-residue decomposition energies and the energy components in terms of the electrostatic (ele) and van der Waals (vdW) interactions for the P1–P9 residues of MICA-TM.

The decomposition (DC) energies were calculated and used to scan for potentially important residues for binding [50]. In order to seek the fingerprint of the HLA/MICA-TM interactions, the interaction energy between each HLA residue and the MICA-TM peptide and *vice versa* was calculated over 100 snapshots of the production phase. The obtained DC energies of the HLA and MICA-peptide residues are plotted in Figs 3 and 4, respectively. The HLA/MICA-TM interactions mostly occurred on the *α1* and *α2* helixes with additionally some residues of *β-*strands (Fig 3). Using the criterion of a total DC energy < -0.5 kcal/mol as an important residue, then 22, 18, 16 and 14 potentially important residues of the HLA-B*51:01, B*35:01, A*26:01 and A*11:01 alleles (S2 Table), respectively.

Van der Waal (vdW) interactions were found to play an important role in the complex, where the magnitude of the MICA-TM peptide binding with the BD-associated HLA alleles (B*51:01 and A*26:01) was greater than that for the corresponding matched non-BD-associated HLA alleles (B*35:01 and A*11:01). For the nonapeptide, as expected the non-polar residues interacted with HLA through vdW interactions (Fig 4). Based on the definition of a total DC energy of < -3 kcal/mol for strong binding, there were four peptide residues that firmly bound to HLA-B*51:01 (P3, P5, P8 and P9) and A*26:01 (P2 and P7–P9), while only two and three residues were found in B*35:01 (P7 and P8) and A*11:01 (P2, P7 and P8), respectively. Note that the P9 and P2 or its adjacent residue are known as the anchor for the peptide accommodation at the binding groove of HLA class I [30, 31]. Loss of the P9 contribution may lead to an unbinding recognition of the non-BD-associated HLAs towards the MICA-TM peptide. More details of the interaction and orientation of this peptide at the binding groove of the four HLA alleles investigated are discussed in the hydrogen bond pattern section.

### H-bond patterns between HLA and the MICA-TM nonapeptide

**Fig 5:**
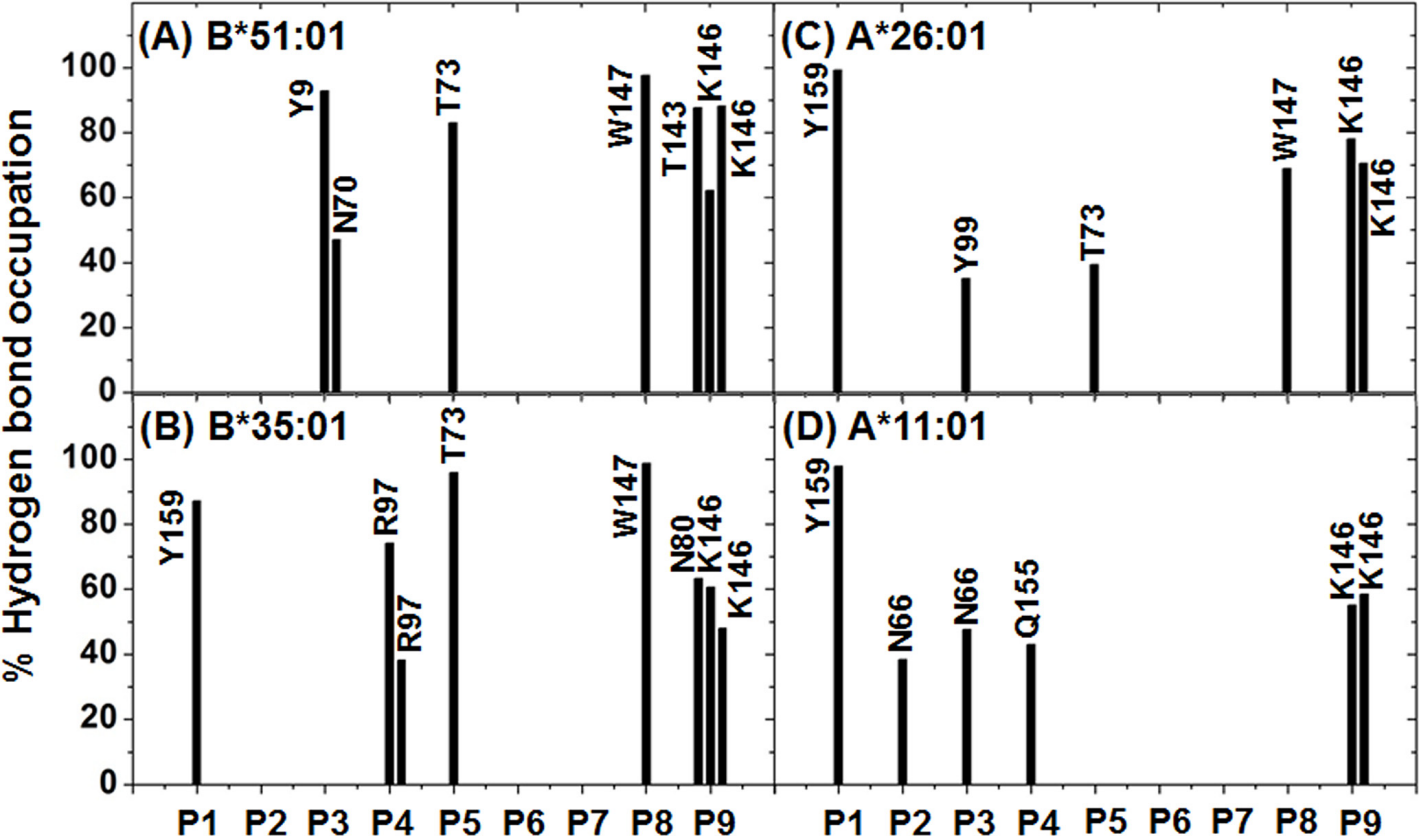
Hydrogen bond interactions. The percentage occupancy of H-bonds averaged over the last 25 ns of simulation time between the nine residues (P1–P9) of the MICA-TM peptide and the HLA residues for the four complexes.

To investigate the intermolecular H-bond interactions between the HLA protein and the incoming short MICA-TM nonapeptide, the number of H-bonds was evaluated using the two acceptance criteria of (i) a distance between the proton donor (D) and acceptor (A) atoms of D−A ≤ 3.5 Å; and (ii) an angle of D−H^…^A > 120°, as previously reported [50, 51]. The obtained results were compared between the two paired BD-associated and non-associated HLA alleles in each locus (A or B), and are shown in Fig 5. The peptide binding sub-sites (S1–S9) were classified by the peptide contact position in the binding groove of HLA, as seen in the HLA-B*51:01/peptide complex (Fig 1D). The last snapshot of each complex was used to represent the protein-protein H-bond formation detected from the MD simulations (see in S2 Fig).

As shown in Fig 1D, the peptide binding groove of HLA class I is constructed by at least five sub-pockets with a large groove volume (S1 and S2 yellow; S3 magenta; S4–S6 dark blue; S7 orange; S8 and S9 light blue), and it is able to support a variety of incoming short peptides in the binding step. Moreover, the binding groove of the HLA protein provides a hydrophobic cavity to support the nine spanning residues (AAAAAIFVI) of the MICA-TM peptide. Due to the nonpolar nature of this peptide, H-bond formation with the HLA protein was expected through the peptide backbones. In Fig 5A and 5B, both HLA-B alleles similarly stabilized the P9 residue of the MICA-TM peptide by the formation of three H-bonds which two of them are the salt bridge interactions between the C-terminal carboxylate group and the ammonium group of K146. The C-terminal residue (P9) could bind significantly stronger with the BD-associated HLA allele than the non-associated allele (~60–90% H-bond occupation compared to only ~50–70% in the non-associated allele), which could be due to the different residue 80 on the *α*1 helix (I80 in HLA-B*51:01 and N80 in HLA-B*35:01). Through the strong H-bond (70% occupancy) with the amide group of N80 (Fig 5B), this polar residue at the S9 sub-site of the peptide induced the C-terminal to change its orientation and move closer to the *α*1 helix of the non-associated BD HLA-B*35:01 (S2 Fig) with the consequence of weakened H-bonds with the *α*2 helix from the loss of interaction with T143 and a decreased interaction with K146. With respect to the N-terminus of the MICA-TM peptide, the Y159 residue on the *α*2 helix of the HLA-B*35:01 allele strongly interacted with the P1 residue (∼90%), whilst this end of the peptide had no interaction with any HLA-B*51:01 residues but instead the P3 residue was stabilized by two *α*1-helix residues, Y9 and N70, with ∼90 and ∼50% H-bond occupations, respectively (Fig 5A and S2 Fig). The rearrangement of the H-bonding network at the N-terminal was previously observed in the octamer and nonamer peptides binding to HLA-B*51:01 [38]. In addition to the protein-protein interactions at the two peptide ends, a strong H-bond was formed between the backbone of the P5 Ala residue and the side chain of the T73 residue on *α*1 helix in the P4–P6 pocket in both HLA-B alleles (> 80%, Fig 5A and 5B). This is congruent with the previous observation that the P5 residue of HIV epitopes (Ile, Val and Pro) was found as an unexpected anchor deeply pointing into the sub-pocket of the binding groove of HLA-B*51:01 [38]. In addition, these two HLA-B alleles had a strong H-bond between the P8 backbone of the MICA-TM nonapeptide and the W147 indole ring on the *α*2 helix.

In the case of the two HLA-A alleles (Fig 5C and 5D), the N- and C-terminal regions of the MICA-TM peptide showed a firmly established interaction, P1 with the Y159 phenyl ring (~100%) and P9 formed two H-bonds or salt bridge interactions with the K146 ammonium group (~70–80% in the BD-associated HLA-A*26:01, but < 60% in the non-associated one), respectively, at the *α*2 helix. Only HLA-A*26:01 (Fig 5C) contained a strong interaction at the nearly C-terminal end (P8 residue) with the *α*2 helix residue W178 (~70%) and the anchor P5 residue weakly interacted with the *α*1 helix residue T73 (~40%). The Y99 residue on the *β*-sheet of HLA-A*26:01 somewhat stabilized the P3 residue of MICA-TM (~40%), while the *α*1 helix residue N66 in HLA-A*11:01 supported the P2 and P3 residues (~40–50%). It is worthy to note that the high protein flexibility of HLA-A*11:01 (Fig 2D) had consequently led to a low binding of the incoming peptide, and in particular at the P5–P9 residues.

Based on the formed H-bonds, the peptide binding recognition was better distinguished in the HLA-A alleles. The lowered binding strength at the C-terminal P9 residue observed in HLA-B*35:01 was suspected to be the most important reason for the low selective binding affinity of this non-BD-associated HLA-B*35:01 allele. Thus, the relatively high protein flexibility in the non-associated HLA-A*11:01 allele led to a decreased H-bond strength with the P9 residue and no stabilization for the P5 and P8 residues. All simulations fairly agreed with the crystal structures of the HLA/peptide complexes in which this P6 residue outwardly located off the binding groove [31].

### HLA/peptide binding affinity

To determine the MICA-TM peptide binding strength towards the four studied HLA alleles, the molecular mechanic/Poisson-Boltzmann surface area (MM/PBSA) approach was applied on the same set of 100 snapshots taken from the production phase. The MM/PBSA approach has been extensively used to predict the overall Gibbs free energy (Δ*G*_*bind*_) in biomolecular systems [52]. The Δ*G*_*bind*_ of the HLA alleles with the peptide bound at the binding groove was calculated from a summation of the total MM energy (Δ*E*_*MM*_), the solvation free energy (Δ*G*_*sol*_) and the entropic term (TΔ*S*). The last term was estimated from the normal mode analysis [53]. Although the Δ*E*_*MM*_ components suggested that the short peptide was better stabilized by the non-BD-associated HLAs in the gas phase, the large destabilization from the solvation effect on the HLA/peptide complex led to a lower total Δ*G*_*bind*_. Rather, within each paired HLA-I gene (HLA-A or HLA-B), the MICA-TM peptide interaction was stronger with the BD-associated allele, where B*51:01 (-56.10 kcal/mol) > B*35:01 (-48.88 kcal/mol) and A*26:01 (-42.97 kcal/mol) > A*11:01 (-31.14 kcal/mol) in Table 1. The obtained results from the present study help to differentiate the HLA alleles and explain a source of BD.

**Table 1:** The binding free energy and energy components (kcal/mol) for the four HLA/MICA-TM complexes predicted by the MM/PBSA method.

	HLA-B*51:01 ^*a*^	HLA-B*35:01 ^*b*^	HLA-A*26:01 ^*a*^	HLA-A*11:01 ^*b*^
Δ*E*_*vdW*_	-74.7 ± 4.2	-78.1 ± 4.7	-80.0 ± 4.8	-63.7 ± 4.2
Δ*E*_*elec*_	-168.4 ± 16.4	-199.4 ± 17.1	-127.2 ± 20.8	-208.1 ± 25.3
Δ*E*_*MM*_	-243.1 ± 16.1	-277.6 ± 16.4	-207.3 ± 21.6	-271.7 ± 25.0
Δ*G*_*polar*_	199.1 ± 13.9	241.1 ± 14.8	177.3 ± 20.4	252.2 ± 23.6
Δ*G*_*non-polar*_	-12.1 ± 0.4	-12.5 ± 0.3	-13.0 ± 0.3	-11.6 ± 0.4
Δ*G*_*sol*_	187.0 ± 13.9	228.7 ± 14.8	164.3 ± 20.4	240.6 ± 23.4
-TΔ*S*	9.7±25.8	12.7±24.1	21.5±28.6	15.4±21.4
Δ*G*_*bind*_	-46.4	-36.2	-21.5	-15.7

HLA alleles are

^*a*^ associated or

^*b*^ not associated with Behçet’s disease (BD).

Data are shown as the mean ± SD, derived from independent simulations. Means within a paired row (HLA-A or HLA-B alleles that are associated with BD versus that are not) followed by a different letter are significantly different.

Δ*G*_*bind*_ is the binding energy with inclusion of entropic term.

### Conserved interaction of the BD associated HLAs

Based on MM/PBSA method, the alanine scanning mutagenesis commonly used to signify the important function of residues was carried out on the two BD associated HLA residues within the 5 Å sphere of the MICA-TM peptide. The results of the relative binding free energy (*ΔΔG*_*binding*_ = Δ*G*_*wild-type*_ *−* Δ*G*_*mutant*_) are given in Table 2. Note that the entropy term of the complex is not significantly changed by only one residue substitution with alanine; therefore the entropic calculation was neglected. By a mutation to alanine on the 40 residues, the HLA residue numbers 70, 73, 99, 146, 147 and 159 contributing the ΔΔ*G* value < -2 kcal/mol in either HLA-B*51:01 or HLA-A*26:01 (Table 2) were considered as the important residues. These six residues could provide the conserved interaction of both BD associated HLAs for binding of the incoming MICA-TM.

**Table 2:** Relative binding free energy upon alanine mutation (ΔΔ*G*_*binding*_) for the HLA residues within 5 Å sphere of the MICA peptide. **The residues with ΔΔ*G***_***binding***_ **of < -2 kcal/mol for both HLA classes are shown in bold text.**

	ΔΔ*G*_*binding*_ (kcal/mol)	
residue	HLA-B*51:01	HLA-A*26:01
Y7	-0.43	-2.40
Y9	-1.49	-0.06
I66/N66	-1.89	-1.90
**N70/H70**	-3.37	-2.57
**T73**	-2.42	-2.45
Y74/D74	-1.67	-0.94
N77	-1.68	-0.73
I80/T80	-1.31	-0.57
A81/L81	0	-0.93
Y84	-0.53	-1.14
Y85	-1.01	-0.11
W95/I95	-1.04	-0.16
T97/R97	-0.08	-3.42
**Y99**	-2.52	-2.22
Y116/D116	-0.99	-1.07
Y123	-1.65	-1.19
T143	-2.25	-1.44
**K146**	-3.68	-2.08
**W147**	-5.54	-4.64
E152	-1.76	-0.82
Q155	-0.15	-2.12
L156/W156	-1.04	-3.44
**Y159**	-3.63	-2.85
W167	-0.03	-1.82

## Conclusions

BD is a multi-organ inflammatory disorder with vasculitis, in which the main cause and mechanism of action are still not well understood. From clinical investigations, the HLA-B*51:01 and HLA-A*26:01 with MICA*009 containing the nonamer peptide (AAAAAIFVI or MICA-TM) are most frequently detected in BD patients. Herein, we aimed to search for the selective correlation between the two BD-associated HLA alleles (HLA-A*26:01 and HLA-B*51:01) and the MICA-TM peptide in comparison with two class matched non-BD-associated HLA alleles (HLA-A*11:01 and HLA-B*35:01) by MD simulations. From the simulations, more contact residues at the binding groove of the BD-associated HLA alleles stabilized the incoming MICA-TM peptide than at the non-associated alleles of the same class (22 and 16 residues for B*51:01 and A*26:01; 18 and 14 residues for B*35:01 and A*11:01). The vdW force was found to be the main protein-protein interaction, but in addition strong H-bonds (>70% occupation) were likely formed with the backbone of the nonpolar peptide, and these were stronger in the BD-associated HLA alleles. The P2/P3 and P9 residues (close to and at the peptide ends, respectively) acted as the anchor for the peptide accommodation at the binding groove of the BD-associated HLAs. The total binding free energy of the HLA/peptide complex suggested a significantly stronger binding strength of the MICA-TM peptide with the BD-associated HLA alleles in the same class (B*51:01 > B*35:01 and A*26:01 > A*11:01). The residues 70, 73, 99, 146, 147 and 159 provided the conserved interaction of the two BD-associated HLAs with the MICA-TM peptide. All the structural, dynamics and energetics information somewhat explain the recognition and selective binding of the MICA-TM peptide towards the specific HLAs related to the BD pathogenesis.

## Supporting Information

S1 Fig Stability of the HLA/MICA-TM complexes. Root-mean square displacements (RMSDs) of all atoms relative to those of the initial structure for the HLA/MICA-TM complex, peptide binding groove, *ß*_2_-microglobulin and MICA-TM peptide in the (A) B*51:01, (B) B*35:01, (C) A*26:01 and (D) A*11:01 HLA alleles bound to the MICA-TM peptide.
(TIF)

S2 Fig Hydrogen bond interactions (dashed line) in the HLA/MICA-TM complexes. The MICA-TM peptide and HLA residues at the binding groove are shown in green and white sticks.
(TIF)

S1 Table Crystal structures used for simulation and number of reported BD patients in association and non-association with HLAs. (DOCX)

S2 Table HLA residues with a total decomposition (DC) free energy (Δ*G*_residue_) of less than -0.5 kcal/mol. (DOCX)
