# Supplementary figures and images for: Molecular Dynamics Simulation Reveals the Selective Binding of Human Leukocyte Antigen Alleles Associated with Behçet's Disease
Source: PLoS One. 2015 Sep 2;10(9):e0135575. doi: 10.1371/journal.pone.0135575 (PMC4557978; doi:10.1371/journal.pone.0135575)

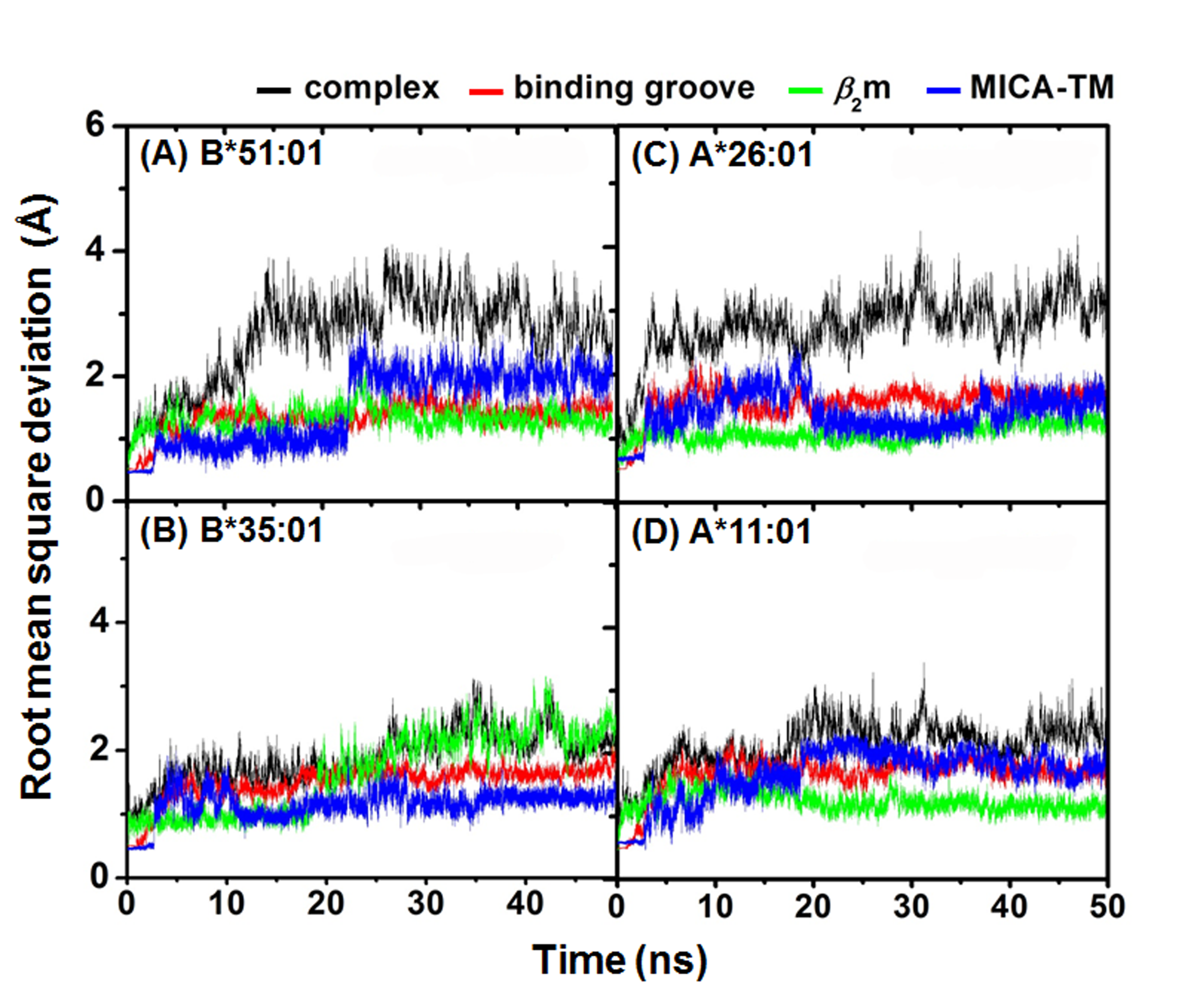

Supplement: S1 Fig — Root-mean square displacements (RMSDs) of all atoms relative to those of the initial structure for the HLA/MICA-TM complex, peptide binding groove, ß 2-microglobulin and MICA-TM peptide in the (A) B*51:01, (B) B*35:01, (C) A*26:01 and (D) A*11:01 HLA alleles bound to the MICA-TM peptide. (TIF) [file pone.0135575.s001.tif]

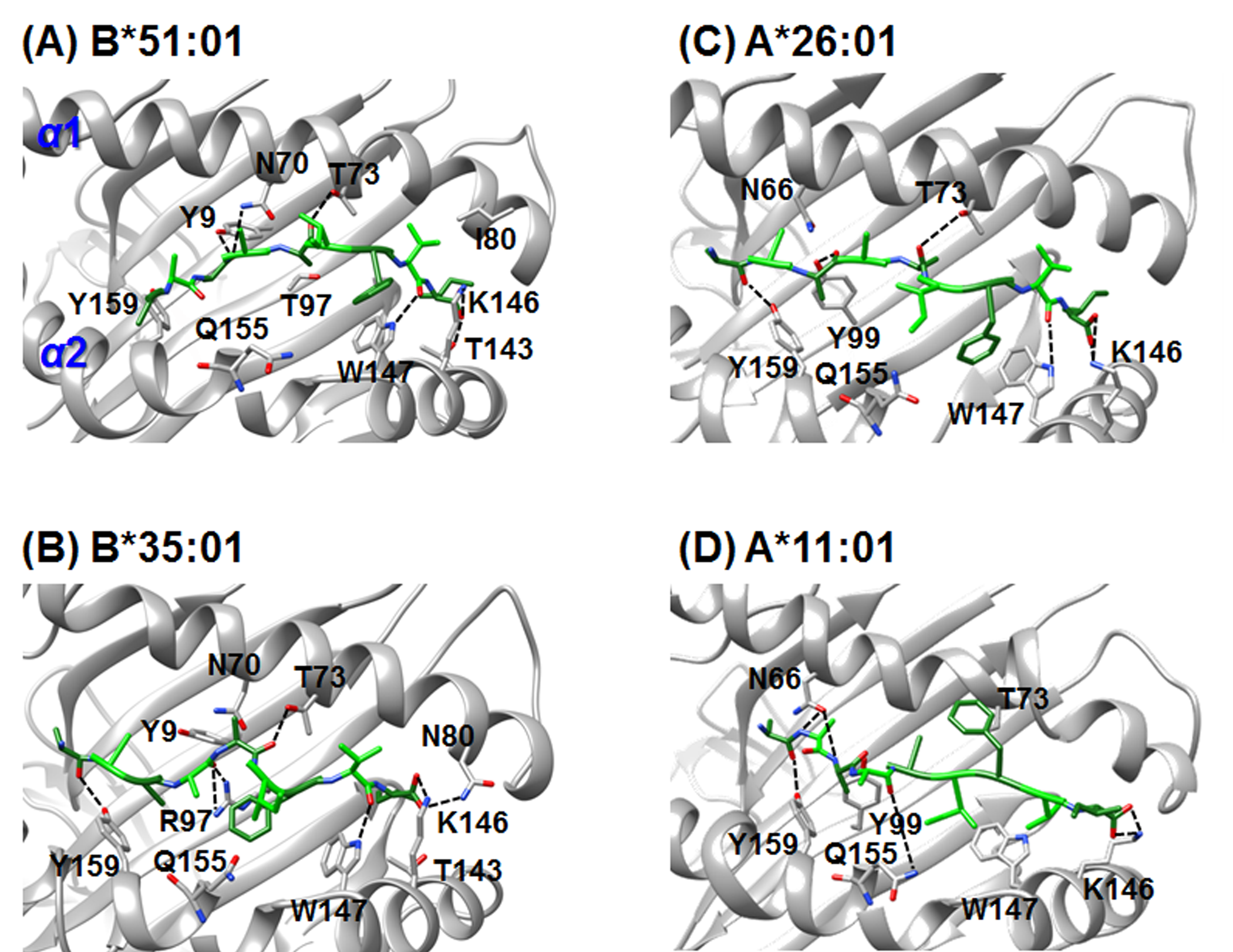

Supplement: S2 Fig — The MICA-TM peptide and HLA residues at the binding groove are shown in green and white sticks. (TIF) [file pone.0135575.s002.tif]
