# Supplementary material for: Molecular Dynamics Simulation Reveals the Selective Binding of Human Leukocyte Antigen Alleles Associated with Behçet's Disease
Source: PLoS One. 2015 Sep 2;10(9):e0135575. doi: 10.1371/journal.pone.0135575 (PMC4557978; doi:10.1371/journal.pone.0135575)
Supplement: S1 Table — (DOCX) [file pone.0135575.s003.docx]

**S1 Table. Crystal structures used for simulation and number of reported BD patients in association and non-association with HLAs.**

|  | **HLA alleles** | | | |
| --- | --- | --- | --- | --- |
| **Experiment** | **Associated with BD** | | **Non-associated with BD** | |
|  | **B*51:01** | **A*26:01** | **B*35:01** | **A*11:01** |
| Crystal structure used in this work | 1E27 [1] | 1X7Q [2] | 1A9E [3] | 1X7Q [2] |
| no. of patients reported for HLA associated and non-associated with BD from 300 cases [4] | 188 | 69 | 46 | 39 |

[1] Maenaka K, Maenaka T, Tomiyama H, Takiguchi M, Stuart DI, Jones EY. Nonstandard peptide binding revealed by crystal structures of HLA-B*5101 complexed with HIV immunodominant epitopes. J Immunol. 2000;165(16):3260-7.

[2] Blicher T, Kastrup JS, Buus S, Gajhede M. High-resolution structure of HLA-A*1101 in complex with SARS nucleocapsid peptide. Acta Crystallogr D Biol Crystallogr. 2005;61(Pt 8):1031-40.

[3] Menssen R, Orth P, Ziegler A, Saenger W. Decamer-like conformation of a nona-peptide bound to HLA-B*3501 due to non-standard positioning of the C terminus. J Mol Biol. 1999;285(2):645-53.

[4] Meguro A, Inoko H, Ota M, Katsuyama Y, Oka A, Okada E, et al. Genetics of Behçet disease inside and outside the MHC. Ann Rheum Dis. 2010;69(4):747-54.
