# Supplementary material for: Molecular Dynamics Simulation Reveals the Selective Binding of Human Leukocyte Antigen Alleles Associated with Behçet's Disease
Source: PLoS One. 2015 Sep 2;10(9):e0135575. doi: 10.1371/journal.pone.0135575 (PMC4557978; doi:10.1371/journal.pone.0135575)
Supplement: S2 Table — (DOCX) [file pone.0135575.s004.docx]

**S2 Table. HLA residues with a total decomposition (DC) free energy (∆*G*_residue_) of less than -0.5 kcal/mol.**

| **B*51:01 *^a^*** | | **B*35:01 *^b^*** | | **A*26:01 *^a^*** | | **A*11:01 *^b^*** | |
| --- | --- | --- | --- | --- | --- | --- | --- |
| **residue** | **∆*G*_residue_** | **residue** | **∆*G*_residue_** | **residue** | **∆*G*_residue_** | **residue** | **∆*G*_residue_** |
| ***α*1 helix** |  |  |  |  |  |  |  |
| I66 | -1.69 | I66 | -2.06 | Y59 | -0.62 | E63 | -3.33 |
| T69 | -0.71 | T69 | -0.68 | N63 | -2.82 | N66 | -0.72 |
| N70 | -1.84 | N70 | -0.54 | H70 | -2.89 | T73 | -1.58 |
| T73 | -2.19 | T73 | -1.91 | T73 | -1.79 | D77 | -0.76 |
| Y74 | -0.66 | S77 | -2.89 | N77 | -0.90 |  |  |
| E76 | -0.55 | N80 | -4.53 | T80 | -0.78 |  |  |
| N77 | -0.93 | L81 | -0.92 | L81 | -0.93 |  |  |
| I80 | -1.46 |  |  | Y84 | -0.76 |  |  |
| Y84 | -0.84 |  |  |  |  |  |  |
| ***α*2 helix** |  |  |  |  |  |  |  |
| I142 | -0.51 | T143 | -1.08 | T143 | -0.78 | K146 | -3.19 |
| T143 | -1.75 | K146 | -3.33 | K146 | -3.96 | W147 | -2.40 |
| K146 | -4.57 | W147 | -3.58 | W147 | -3.17 | A150 | -0.63 |
| W147 | -3.02 | V152 | -1.49 | W156 | -2.38 | A152 | -0.68 |
| A150 | -0.71 | L156 | -1.29 | Y159 | -1.91 | Q155 | -0.54 |
| Q155 | -0.52 | Y159 | -2.67 | W167 | -2.70 | Y159 | -2.51 |
| L156 | -1.16 | W167 | -2.17 |  |  | W167 | -1.69 |
| Y159 | -3.72 |  |  |  |  |  |  |
| L163 | -0.71 |  |  |  |  |  |  |
| ***ß*-strand** |  |  |  |  |  |  |  |
| Y9 | -0.64 | Y7 | -1.67 | Y7 | -1.46 | Y7 | -2.24 |
| W95 | -0.55 | R97 | -2.34 | Y99 | -1.87 | Y9 | -0.63 |
| Y99 | -2.03 | Y99 | -2.38 |  |  | Y99 | -2.89 |
| Y123 | -0.54 | Y123 | -0.54 |  |  |  |  |

HLA alleles are *^a^* associated or *^b^* not associated with Behçet’s disease (BD)
